# Starting a Research Career in Cardiology: Advice for Fellows in Training and Early-Career Cardiologists

**DOI:** 10.14797/mdcvj.1108

**Published:** 2022-06-03

**Authors:** Nino Isakadze, Francoise A. Marvel, Yvonne Commodore-Mensah, Seth S. Martin, Erin D. Michos

**Affiliations:** 1 Ciccarone Center for the Prevention of Cardiovascular Disease and Johns Hopkins University School of Medicine, Baltimore, Maryland, US; 1 Johns Hopkins Center for Health Equity and School of Nursing, Johns Hopkins University School of Medicine, Baltimore, MD, US; 3 Ciccarone Center for the Prevention of Cardiovascular Disease, and Johns Hopkins Center for Health Equity at Johns Hopkins University School of Medicine, Baltimore, Maryland, US

**Keywords:** academic cardiology career, mentorship, sponsorship

## Abstract

Launching an academic career in cardiology can be challenging. Mentorship has long been considered a core component in the academic career advancement of trainees across different disciplines and career stages, including cardiovascular disease. But simply having a mentor may not be sufficient to embark on a successful academic journey in cardiology. In this paper, we share advice on starting a research career in cardiology from both the mentee and mentor viewpoints. These perspectives reflect academic career guidance models developed at the Johns Hopkins Center for Mobile Technologies to Achieve Equity in Cardiovascular Health, which is funded by an American Heart Association Strategic Focused Network grant, to emphasize training. Core principles include encouraging mentees to develop a unique professional identity cultivated by a diverse, collaborative, and effective mentorship and sponsorship team.

## Introduction

Cardiovascular disease remains the leading cause of morbidity and mortality in the United States, requiring continued academic productivity to improve cardiovascular health care through research and innovation.^1^ However, early-career cardiologists often encounter challenges when launching an academic career, including lack of sustainable funding, a competitive funding landscape, funding competition with PhDs, insufficient protected time, increased clinical requirements, and work/life balance issues.^2^ These challenges may even cause early-career cardiologists to avoid or leave the academic workforce.^3,4^ During this critical time in one’s career, it is essential to have an effective support system and access to institutional or noninstitutional resources to overcome potential barriers.

Mentorship has long been recognized as an essential component for trainee personal development, academic career advancement, satisfaction, and research development.^2,5^ However, two independent surveys of members of the American College of Cardiology (ACC) show that mentorship alone may no longer be associated with objective academic success.^2,3,6,7,8,9,10^ While more studies are needed to further evaluate the relationship, it raises concerns that not all mentorship is the same and that effective mentorship may be declining. Another concern is that without concurrent sponsorship, mentorship alone is insufficient for launching a successful academic career. Sponsorship can be especially meaningful during training and early-career stages to facilitate networking and identify funding opportunities, which may lead to promotion of a successful academic career. Other components of starting a successful academic cardiology career include resources available to trainees at their institution, personal drive, collaboration, and availability of early-career bridge funding.

In this article, we share professional experiences from both the mentor and mentee perspectives on how to launch a successful academic career and overcome common barriers (***Table 1***). As an example, we use training models implemented at our Johns Hopkins Center for Mobile Technologies to Achieve Equity in Cardiovascular Health. This center was funded by an American Heart Association (AHA) Strategic Focused Research Network (SFRN) grant in Health Technology and Innovation,^11^ which has a core emphasis on training the next generation of a diverse biomedical workforce in cardiovascular disease research.

**Table 1:** Essentials of success in a research career.


ESSENTIALS OF SUCCESS IN A RESEARCH CAREER

Finding passion, niche and research focus

Finding an effective mentor

Finding an effective sponsor

Seeking peer support and junior mentorship

Creating an individualized development plan

Engaging in career development activities

Applying for and obtaining grant funding

Disseminating work via publications, presentations, and social media


## Motivation and Support

Starting a cardiology fellowship is an exciting career phase that brings new experiences and challenges. Fellows strive for clinical excellence and, at the same time, try to find time and space for academic pursuits. The beginning of fellowship training is an excellent time to reflect and clarify one’s area of interest and passion. Thinking about *why* one is choosing a specific research space may help increase motivation while also helping to identify research ideas. Connecting with other trainees and faculty members (either within or outside cardiology) to discuss personal interests may guide one to further refine their ideas. Contrary to what many trainees believe, it is not necessary to have a clearly outlined research question, study design, and analysis plan prior to approaching senior colleagues for advice or potential mentorship. However, starting this process early is important to prevent delays in research activities due to unforeseen circumstances posed by regulatory, funding, or clinical reasons.

**Figure 1:**
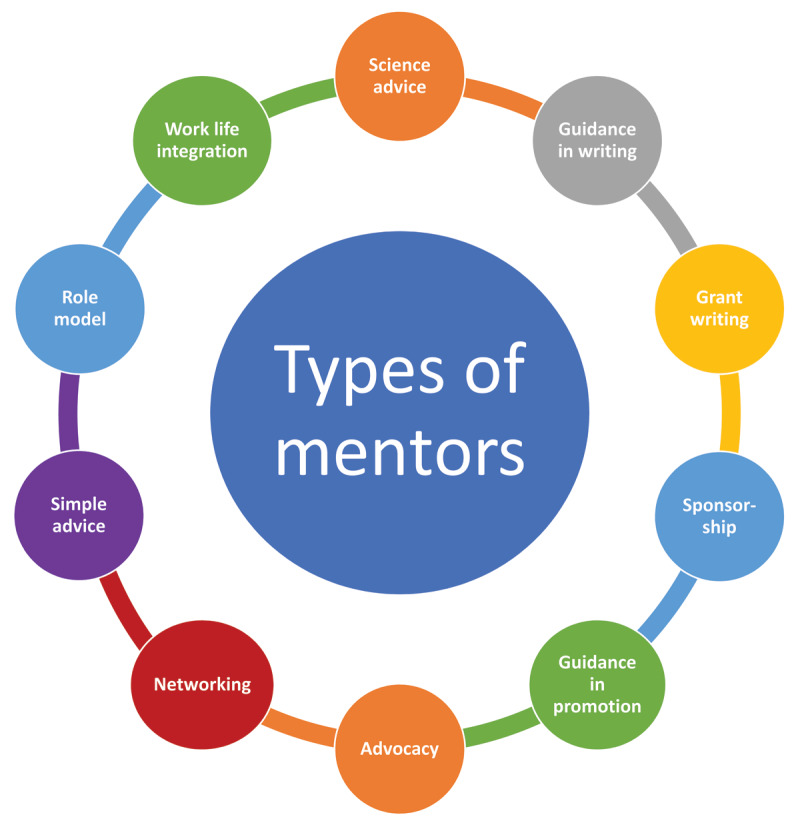
Various mentor types.

Mentorship is a critical component for success in academic medicine. The word mentor means “a trusted counselor or guide” and typically implies a relationship that is built over time. There are different types of mentors. For example, research mentors can help guide scientific and conceptual development in a field of work, while career mentors can help guide academic and career development. Additional mentors can provide guidance on work-life integration and serve as role models. A mentor can take on one or more of these roles. Mentees may have both formal and informal mentors with varying time commitments. It is unlikely that one single person can fulfill all the mentoring roles needed for a given individual, thus a mentoring team is often necessary for success in launching a research career (***Figure 1***). Note that mentoring is important at all stages of one’s career and not limited to those in their early careers. As a trainee or an early-career faculty progresses through their career, the mentoring relationship and mentoring team will naturally evolve as well.

Once a mentoring relationship is established, it is helpful to schedule standing one-on-one meetings. The frequency of these meetings depends on the goals for the relationship; the primary research advisor should spend sufficient time to ensure that the mentee is progressing towards their goals. In turn, the mentee should take initative in these meetings and come prepared with an outline of what they would like to discuss. If feasible, having a network of junior mentors and colleagues that are all connected to the same mentor (ie, a lab group team) is ideal to provide even more support. Note that in this era of digital technology and virtual meeting platforms, mentors no longer must be limited to those within the “brick and mortar” of one’s own institution but can be found across the country and globally. Indeed, with sufficient planning and dedication, all of the principles of effective mentorship can be achieved through outside institutions.

If new to an institution and unsure of where to find a mentor, the fellowship program director, division chief, department chair, or other senior faculty can help identify faculty in one’s preferred area of research interest who may be a potential mentor. Mentees may then reach out to those individuals to discuss interests and explore a potential fit. Although formal mentorship or pairing programs can be helpful, most effective mentee/mentor relationships are formed when both people have aligned interests, mutual respect, and understanding. The most effective mentoring relationships are bidirectional, where both parties mutually benefit from working together. Discussing one’s passion and research interests often clarifies whether a successful mentor/mentee relationship may be formed. The mentee also should inquire about the experiences and career trajectories of other mentees working with their potential mentor. This offers insight into whether a mentor has an interest in supporting the mentee and their career goals, has a strong academic track record in one’s field of interest, is responsive, and has time to provide necessary guidance. They also should value collaboration and be willing to connect their mentee with individuals and resources necessary for career success.

In addition to a mentor, trainees and early-career faculty benefit from having one or more sponsors. A sponsor is a person in a position of influence and power who has access to networks and resources and who actively supports the career of a protégé who they believe has potential. Note that a mentor also can be a sponsor if in a position of power and influence, but not all mentors may be equipped to be sponsors. Progress in a faculty career depends on having one or more advocates who will put their mentee’s name forward for professional opportunities. However, some senior faculty who have the capacity to sponsor may be unaware of a trainee or early-career faculty’s research interests. Mentees must be proactive in reaching out to such individuals and expressing their interest in such opportunities when they arise. Networking opportunities, such as those that take place at professional society meetings such as the AHA or ACC Scientific Sessions, are good places to foster these introductions.

Another recommendation is to look at one’s research through the lens of health equity.^12^ This includes seeking mentors, sponsors, and/or collaborators with specific expertise and experience in health equity and finding training opportunities at research centers that focus on health equity. It also is advisable to seek mentors who help build equitable and inclusive cultures with trainees and collaborators who are demographically diverse and from a variety of disciplines.

The value of peer support may be underrecognized. Co-fellows, fellow mentees, and peer early-career faculty members may provide timely and valuable advice on one’s journey. Sharing experiences on different career development opportunities, navigating relationships in an academic career, and managing work/life balance may be among the important components in promoting well-being and confidence in career decisions. Cardiology fellows and early-career faculty should consider mentoring more junior postdoctoral fellows, residents, or students, which can be mutually beneficial. Fellows who mentor will learn how to develop as mentors themselves while learning from mentees how to be a more effective mentee. Anyone with an interest in serving as a mentor can do so since there is always someone aspiring to reach the next professional level and learn from a predecessor’s journey.

## Career Development Activities and Career Trajectories

One of the most challenging times in one’s academic medicine career is the transition from fellowship to faculty. Early-career cardiologists are typically defined as those within the first 10 years of completing a cardiology fellowship training program. There are several different pathways in academic cardiology, ranging from physician-scientist, clinician educator, clinician administrator, and pure clinician to variations among these roles. The amount and type of research to pursue depends on one’s career goals and interests; faculty start-up packages often base their protected research time on the expectation that the early-career faculty shows high potential for achieving grant funding to support their time. Many challenges and uncertainties can push early-career cardiologists away from academic medicine into other career pathways (ie, private practice, industry, etc). These challenges are heightened by declining rates of grant funding success, increasing clinical demands in response to declining clinical reimbursement, more time needed to maintain clinical competency, financial pressures (such as overreliance on relative value units, or RVU, models for bonuses that reward clinical activity and discourage research), lack of RVU credit for academic pursuits, and disadvantages when competing with PhDs for grant funding, among others.^4^ Surveys from the ACC also indicate insufficient mentorship, insufficient collaborators, lack of bridge funding, and insufficient time during work hours as major obstacles to pursuing academic and research actitivies.^4^ Conversely, effective mentorship, sponsorship, collaboration, and bridge funding for protected time can be instrumental for retaining talented individuals in academic medicine; these protective factors should be cultivated at an institutional level.

Depending on previous training, competency, and available time, trainees may benefit from completing coursework related to their interests during their fellowship and early career. These may be single or multiple courses in statistics or epidemiology, design, artificial intelligence, or clinical trials at one’s institution, an outside institution, or online. Many institutions offer courses in grant writing and other professional development topics. Trainees also may consider volunteering by joining national cardiology society councils and sections as well as writing groups, peer reviewing papers, and moderating. It also is helpful to look for funding opportunities designed for fellows and early-career cardiologists, starting with local foundations and even federal or industry funding.

Industry collaboration is another valuable pathway for funding and partnership opportunities that support early-career innovative research. For example, Apple provides investigator support opportunities for device donations and support of digital health initiatives. Using an example from the authors of this paper, a mentee and her primary mentor collaborated with Apple based on their shared vision of optimizing secondary prevention through technology tools that empower and engage patients diagnosed with acute myocardial infarction. Through partnership and support from Apple, they designed a digital health program and conducted a multisite study supported with an Apple Watch device donation. Their industry collaboration continues today and includes ongoing feedback and support for their digital health research.

Trainees also can consider applying for fellowships such as the AHA SFRN,^11^ which provides a unique collaborative environment for training. Such fellowships enable trainees to take advantage of additional career guidance from each center’s training directors, connect with peers and senior mentors across the nation, and collaborate and establish a presence at the national level.

One helpful tool for trainees and early-career faculty is to draft an individual development plan (IDP) to clarify and define short- and long-term academic goals. Although primarily for personal use in the beginning, it can be updated and reviewed routinely throughout one’s career to track progress. Once a mentoring relationship has been established, the mentee should review the IDP with the mentor/mentoring team to help confirm that both mentee and mentor(s) are aligned on goals and expectations. The IDP should have clearly defined milestones with an achievable timeline.

## Grant Writing and Funding

Many early-career cardiologists carry substantial educational debt, adding financial pressure that may dissuade individuals from pursuing a research career. The National Institutes of Health (NIH) Loan Repayment Program (LRP) is one mechanism that may help recruit and retain individuals with potential to build and sustain a research career. The NIH LRP will cover up to $50,000 annually of the researcher’s educational debt in return for their commitment to conduct research relevant to the NIH mission.^13^ Application for the LRP can occur during fellowship or early career as long as one has sufficient protected time and meets other eligibility criteria.^13^ For the senior author of this paper, the LRP program was pivotal to her success in pursuing a career in academic cardiology following her fellowship despite having substantial educational debt. Fellows-in-training with protected time for research also may be funded by AHA-funded fellowships such as the SFRN, Dorris Duke Physician Scientist Fellowship, Merck Research fellowship, individual F32 fellowships funded by the NIH, or institutional T32 fellowships funded by the NIH.

**Figure 2:**
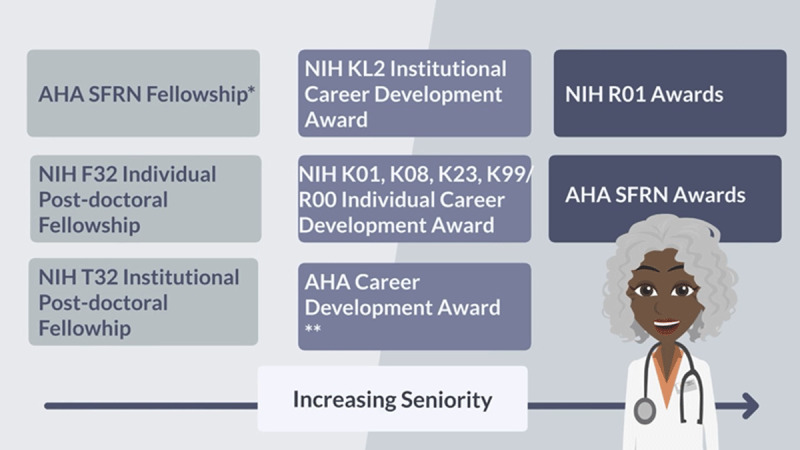
Academic funding opportunities throughout a career. AHA: American Heart Association; SFRN: Strategically Focused Research Network; NIH: National Institutes of Health. * Other post doctoral fellowship awards: eg, Doris Duke Physician Scientist and Merk Research Fellowship Award. **Other awards: eg, Doris Duke Physician Scientist Award, Donaghue Foundation Award, Patient-Centered Outcomes Research Institute Awards.

For early-career faculty, the typical pivotal milestone is the attainment of a career development award such as those funded by the AHA or the NIH-funded K awards (***Figure 2***).^14^ These mentored grants serve to cultivate the development of a promising early-career faculty member towards becoming an independent researcher who would then become competitive for independent grant funding through such mechanisms as the NIH R01 awards. The NIH K awards offer 3 to 5 years of supervised research with the goal of becoming an independent researcher. These career development awards typically stipulate 75% protected time towards research, with some exceptions of 50% protected time for certain surgical or procedurally intensive specialties, such as interventional cardiology or electrophysiology. Briefly, the K23 is a mentored patient-oriented research award geared towards clinical research with direct patient/participant involvement. The K08 is a mentored clinical scientist development award geared towards basic or translational science research. We encourage trainees and early-career faculty to discuss with their mentors whether their current projects are sufficiently preparing them to successfully apply for a career development award grant.

## Dissemination of Work: Publications and Social Media

For those pursuing a clinician-researcher pathway, publications and grants are the “currency” for success in academic medicine and a path towards promotion, so it is important to set aside time for writing. The senior author of this paper blocks out time on her calendar for writing each week, reserving dedicated time that is protected from clinical work, administration, and meetings. Since the most challenging part of writing can simply be to get started, one recommendation is to put ideas down on paper; even if the draft is initially in disarray, it can then be refined. Creating a proposal or outline for the work is a useful starting framework. When writing a scientific paper, this senior author finds it easiest to write the methods and results section first and then the introduction followed by the discussion section last.

For trainees and early-career faculty who committed to writing a paper or doing a project, bringing the work to full completion is important. This may entail planning to avoid over-committing on projects and taking on too much, understanding the time required for each project, and cultivating time management skills. Ideally, every abstract should be converted into a full manuscript. Because original science manuscripts carry the most weight in one’s professional career and help towards grantsmanship, more of one’s time and energy should be spent generating new science. However, when starting out, writing state-of-the-art review articles or editorial/viewpoint articles can help a trainee/early career develop a broader understanding of the context of a field and identify gaps for future research.

Note that a great paper is often rewritten multiple times. A well-presented paper is critical to its likelihood of acceptance in a journal; conversely, poor writing or unclear figures or tables that do not complement the text can detract from an otherwise high-quality paper. Having an outside colleague read the paper is helpful to ensure comprehension and solid methods and results. Authors must be careful about self-plagiarism and ensure that the text included in the manuscript is rewritten for each publication—even when using an established cohort or a previously used methodology—because most journals run iThenticate or similar plagiarism software. When writing a paper, the author must consider what a reviewer may criticize about their work and try to address this before submitting. Original science papers should have a limitation section where the authors can acknowledge these shortfalls before the reviewer identifies them. Cite the most relevant papers, as reviewers will notice major omissions in the field. Importantly, authors should make sure to explicitly address the novelty and impact of their work; even a well-conducted, well-written paper may be rejected by a journal for low priority.

Rejection of a manuscript (or a grant) can be very discouraging. Still, new authors should understand that rejection is a common part of the academic process. Ideally, constructive reviewer comments can be an opportunity to further improve the paper. If worth writing in the first place, every paper has a “home,” and finding the right fit depends on the scope of the journal and whether the research aligns with the journal’s priorities. The peer review process, while generally well-intentioned, can be a fickle and subjective process. One’s work may resonate better with another set of reviewers or at a different journal target, so authors should not lose hope. As basketball star Michael Jordan famously said, “I’ve failed over and over and over again in my life. And that is why I succeed.”^15^ One’s mentor and colleagues can help suggest the next target for one’s work to ultimately result in acceptance.

**Figure 3:**
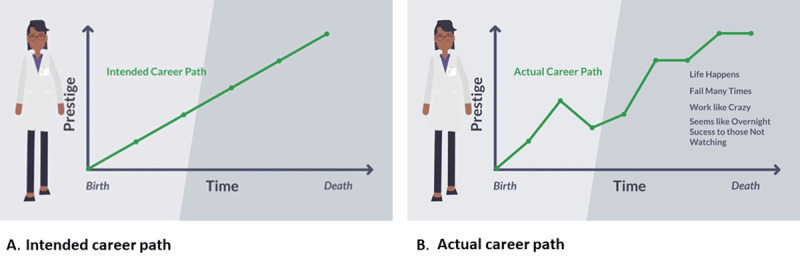
Career progression paths.

Indeed, research success requires hard work, but success also takes focus, support, balance, and sometimes sheer luck. Embrace failure as part of the research process, and know when to keep persisting and when to change directions (one’s mentors and sponsors can provide guidance here). An academic career is a marathon rather than a sprint and often does not proceed in a linear fashion (***Figure 3***)—it takes persistence, perseverance, and grit. However, the discovery of new science, especially as it aligns with one’s passion, can be tremendously rewarding. Additionally, becoming a mentor oneself and supporting the next generation of researchers brings great personal fulfillment.

Engaging in social media platforms such as Twitter can be advantageous for trainees and early-career faculty to advance their research careers by facilitating instantaneous global dissemination of their research and presenting new opportunities for networking and collaborations.^16,17^ Cardiovascular researchers should consider taking advantage of Twitter’s wide reach to begin building their online portfolios, elevate access to their publications, and ultimately advance their careers. Social media can foster improved visibility of one’s work and connect researchers not only to other researchers and clinicians but also to the public, policy makers, and other stakeholders.^16^ Engagement in social media can be an important channel for building community and professional development, particularly for women in cardiology.^18^

Altmetrics are alternative metrics that track the attention a scholarly work receives in nontraditional sources, such as the news, blogs, and social media posts. Studies have shown that articles with higher altmetric scores are more likely to be downloaded and cited, which translates to an increase in traditional academic metrics (ie, citation count, H-index).^17,19^ Indeed, as demonstrated in several randomized trials conducted by academic journals, active promotion of their articles on social media translated into a greater number of downloads and higher citation rates.^20,21,22^ Even if not required by the journal, researchers should strongly consider creating illustrations for their original science, as figures are more likely to be shared on social media and downloaded into PowerPoint talks for greater dissemination. A social media management platform called Buffer found that its posts with images received 150% more retweets than those without.^16^ When sharing one’s work on social media, tweets should include the source reference (ie, the URL to the paper) so that others can easily find the whole article and potentially download it for reading.

## Innovations in Cardiology: A Bright Future for Research

The landscape of cardiovascular disease management is changing, with new opportunities brought on by digital health technologies and the rapid increase in mobile technology ownership.^23,24^ This is an exciting time to participate in the digital transformation of cardiovascular care to improve care delivery and health equity. Through leading projects in digital health, trainees are able to work with their mentors and develop skills in multi- and transdisciplinary team building and project management. Examples of opportunities for research in these area include large data analytics, artificial intelligence, telehealth, digital health intervention design, implementation and evaluation, and more. Through working on innovative health technology projects, trainees also gain an opportunity to learn and develop skills in collaboration with engineers and tech companies; develop patents; and understand the regulatory, insurance, and policy aspects of technology development and implementation. Furthermore, trainees may pursue specific courses in technology design development and prototype development.

## Conclusion

Starting one’s research career during cardiology fellowship training and early career may seem like a daunting task. However, success depends on finding one’s passion, having clearly defined goals set forth in an individualized development plan, having a collaborative and effective mentorship team, and sponsorship. A career in academic cardiology that entails research can be tremendously rewarding and worth the effort.

## Key Points

- Identifying one’s career interest/passion and finding mentors and sponsors is a crucial starting point in early-career development.
- Engaging in various career development activities helps early-career advancement through networking, learning, and collaboration.
- Writing and obtaining various grants and learning skils for disseminating one’s work helps establish an academic career path.
